# The factors that influence oral health-related quality of life in 15-year-old children

**DOI:** 10.1186/s12955-018-0847-5

**Published:** 2018-01-18

**Authors:** Ling Sun, Hai Ming Wong, Colman P. J. McGrath

**Affiliations:** 1 Paediatric Dentistry and Orthodontics, Faculty of Dentistry, The University of Hong Kong, Pokfulam, Hong Kong; 2 Periodontology and Public Health, Faculty of Dentistry, The University of Hong Kong, Pokfulam, Hong Kong

**Keywords:** Oral health-related quality of life, Periodontal status, Caries, Malocclusion, Sociodemographic factors

## Abstract

**Background:**

Several hypotheses on factors that influence oral health-related quality of life (OHRQoL) have been proposed but a consensus has not been reached. This cross-sectional study aimed to analyse the sociodemographic and clinical factors that may influence the OHRQoL of 15-year-old children.

**Methods:**

A representative sample was selected from Hong Kong. Periodontal status and caries were examined according to WHO criteria. Four orthodontic indices were used to assess malocclusion. Child Perception Questionnaire (CPQ_11–14,_ 37 items) including four domains, namely oral symptoms (OS), functional limitations (FL), emotional well-being (EWB), and social well-being (SWB), was used to measure OHRQoL. Adjusted OR was calculated by ordinal logistic regression.

**Results:**

A total of 364 eligible subjects (186 girls, 178 boys) were recruited. The prevalence of caries was higher in girls than in boys (*P* = 0.013). Compared with girls, boys tended to have a better experience in the domains of EWB, SWB and the total CPQ (adjusted OR = 0.46, 0.59 and 0.61, respectively). Unhealthy periodontal conditions were more prevalent than caries (92.6% vs. 52.7%); moreover, periodontal conditions with CPI scores of 2 had a negative effect on the domain of SWB and the total CPQ (adjusted OR = 1.76 and 1.71, respectively). Only the most severe malocclusion showed an effect on the domain of FL and the total CPQ (adjusted OR = 1.55 and 2.10, respectively). Little effect of family ecosocial factors and caries was found on CPQ scores.

**Conclusion:**

In this study, gender, periodontal status, and malocclusion showed an effect on OHRQoL after adjusting for potential confounders. Boys had less caries and better OHRQoL than girls did. Unhealthy periodontal conditions led to worse social welfares and OHRQoL. The most severe level of malocclusion caused oral functional limitations, hence worse OHRQoL.

## Background

Patient-centred treatment requires clinicians to put efforts not only on physical diseases but also on the improvement of patients’ psychosocial well-beings. Hence health-related quality of life, which measures four broad domains, namely physical health, psychological well-being, social relationships, and environment, has become a research focus in recent years [1]. In dentistry, the concept of oral health-related quality of life (OHRQoL) was raised about two decades ago [2].

Currently, several hypotheses on OHRQoL have been proposed. First, health problems may affect quality of life, but such a consequence is not inevitable [3]. For example, an individual who had eating problems due to pain and discomfort would have rated this problem as extremely important at one point of time. However, when this problem was diagnosed as oral cancer, and treated with radiotherapy and/or surgery, the same individual might report the original problem as relatively unimportant [4]. Second, quality of life is a “dynamic construct” that is likely to change overtime [4]. The value attributed to any domain of quality of life may change over the life span [5]. Third, sociodemographic factors may also influence OHRQoL [6, 7]. The nature and magnitude of impacts could vary between populations of different cultural backgrounds [8]. The most frequently investigated factors were gender and family ecosocial factors such as household income and parents’ education [9–11].

Although many studies have been conducted in this research area, a consensus has not been reached [11–17]. This article is a cross-sectional analysis aimed to analyse the clinical and sociodemographic factors that may influence OHRQoL. The sample of this study was randomly selected from 15-year-old students in Hong Kong.

## Methods

### Measurement instruments

Child Perceptions Questionnaire (CPQ_11–14_) with 37 items was used to assess children’s OHRQoL [18–21]. The questionnaire consists of four domains, namely oral symptoms domain (OS, 6 items), functional limitations domain (FL, 9 items), emotional well-being domain (EWB, 9 items) and social well-being domain (SWB, 13 items). Each item has a 5-point response format ranging from 0 to 4. The item scores of each domain are added together to get a domain score, and the scores of four domains are added together to get the total CPQ score. Higher scores represent poorer OHRQoL [22].

Community Periodontal Index (CPI) and the Decayed, Missing and Filled Teeth (DMFT) index were used to measure periodontal and caries conditions according to the criteria of WHO [23]. Also, Significant Caries Index (SiC index) was used to classify caries. Individuals are sorted according to their DMFT values; the one third of the population with the highest caries score is selected and the mean DMFT for this subgroup is calculated; this value constitutes the SiC Index [24].

Index of Orthodontic Treatment Need (IOTN), Dental Aesthetic Index (DAI), Index of Complexity, Outcome and Need (ICON), and Peer Assessment Rating (PAR) were used to assess orthodontic treatment need and complexity [25–30].

IOTN includes Dental health component (DHC) and Aesthetic component (AC). DHC has 5 grades (no need to very great need) and the worst occlusal trait is recorded to allocate the grade. AC is comprised of 10 front view photographs, which represents 10 scales of dental attractiveness. The IOTN (DHC) or IOTN (AC) grading can be further categorized into three orthodontic treatment groups (DHC 1–2 or AC 1–4, no need; DHC 3 or AC 5–7, borderline need; DHC 4–5 or AC 8–10, definite need) [31, 32].

The index of DAI is calculated by multiplying the measurements of 10 occlusal traits by their weights; the addition of their products and the addition of a constant number, 13, is the final DAI score. It can be categorized into 4 scales of orthodontic severity and treatment need (≤ 25, normal or minor malocclusion-no treatment need or slight need; 26–30, definite malocclusion-treatment selective; 31–35: severe malocclusion-treatment highly desirable; ≥ 36: very severe (handicapping) malocclusion-treatment mandatory) [28].

ICON is used to evaluate treatment need, treatment outcome and complexity [29]. Its aesthetic score is assessed using the IOTN (AC). Five occlusal trait scores are multiplied by their respective weights and summed to calculate the ICON score. The ICON score can be scaled into 2 categories for treatment need (≤ 43 No; > 43 Yes), and 5 categories for orthodontic complexity (< 29 easy; 29–50, mild; 51–63 moderate; 64–77 difficult; > 77 very difficult). It puts heavy emphasis on aesthetics.

PAR is an estimate of how far a case deviates from normal. The concept is to assign a score to 11 components of occlusal traits that make up a malocclusion. The individual scores are summed together to obtain an overall total, representing the degree a case deviates from normal occlusion. Generally a measure of 10 or less indicates an acceptable alignment and occlusion, and 5 or less suggests an almost ideal occlusion [27].

### Study population and data collection

This study was part of a longitudinal study that was planned to follow subjects from age 12 to 18. Cluster randomized trial was used in the original design of the study. The sampling frame was all local secondary schools in Hong Kong (by law all children are required to attend secondary school). A random sample of 45 schools (approximately 10% of all local secondary schools) from 18 districts in Hong Kong, SAR, was selected. Students born between April 1st and May 31st, 1997 were invited to participate in the oral health survey conducted by Faculty of Dentistry, the University of Hong Kong. The sample of this study was selected from the birth cohort of “children of 1997” [33].

It should be noted that at age 15, not only subjects who were followed up from age 12 came to the survey again, but also some subjects, who did not show up in the 12-year-old survey, were willing to participate in the 15-year-old survey. Therefore, this article is a cross-sectional analysis of all these 15-year-old subjects; the longitudinal analysis from age 12 to 15 will be demonstrated in another article.

Students’ oral health status was examined using an intra-oral disposable mouth mirror with a built-in LED light source. The same trained and calibrated examiner performed the oral examination according to the criteria of WHO [23]. Front-view dental photos were taken by extracting lips using oral retractors to assess IOTN (AC). Dental impressions were collected and the plaster models were sent to OrthoLab (Poland) to make digital models. Software O3DM (version3.8.5 (c) by OrthoLab, Poland) was used to analyse digital models by the same examiner. Reassessments were performed among 10% randomly selected samples after 2 weeks of first assessment to test intra-examiner’s reliability.

Systematic health information, dental treatment history, family ecosocial factors including father’s education, mother’s education, and household income were collected from a self-completed questionnaire. OHRQoL was assessed by CPQ_11–14_.

Subjects were excluded from the final analysis if they were systemically unhealthy, had orthodontic treatment history, or had oral diseases other than caries, periodontitis and malocclusion.

### Ethics, consent and permissions

The ethical approval of this study was granted by the Institutional Review Board of the University of Hong Kong/Hospital Authority Hong Kong West Cluster (UW 09-453). A written consent from parents/primary caregivers and a verbal consent from students were obtained from all participants.

### Statistical methods

For intra-examiner reliability tests, kappa values were used for CPI, weighted kappa for IOTN (DHC) and IOTN (AC), and Intra-class correlation coefficient (ICC) for DAI score, ICON score, and DMFT.

Mann-Whitney U test was used to analyse whether there was a difference of oral health status between girls and boys; independent samples t test was used to detect the difference of mean DMFT.

The effects of sociodemographic and clinical factors on OHRQoL were analysed with parameters set as follows:

1. Dependent variables: for bivariate analyses, dependent variables were set as the scores of OS, FL, EWB, SWB and total CPQ; for ordinal regression, dependent variables were set by grouping these scores into four ranks with quartile values as cut-off points.
2. Independent variables: gender, father’s education (primary school graduate or below; secondary school, post-secondary or above), mother’s education (primary school graduate or below; secondary school, post-secondary or above), household income (below HK$10000, HK$10001-HK$20000, HK$20001-HK$30000, HK$30001-HK$40000, HK$40001 or above), periodontal status (CPI score < 2, CPI score = 2), caries experience (DMFT < SiC Index value, DMFT > = SiC Index value), and orthodontic treatment need (measured by IOTN, DAI, ICON and PAR).
3. Bivariate analyses: for parametric tests, comparison between two samples used the independent samples t test, others used the one-way ANOVA; for nonparametric tests, comparison between two samples used the Mann-Whitney U test, others used the Kruskal-Wallis H test.
4. Multivariate analyses: ordinal regression (link function: logit; model: main effects) was used to calculate the adjusted odds ratios (OR). The statistical software package SPSS (version 22, SPSS Inc., Chicago, IL, USA) was used for ordinal regression (procedure: Analyse-Regression-Ordinal Regression). To avoid interaction effect, orthodontic treatment needs measured by different orthodontic indices were entered into regression separately. To calculate adjusted OR, malocclusion was adjusted for gender, father’s education level, mother’s education level, household income, periodontal status, and caries experience; while gender, socioeconomic status, periodontal status and caries experience were adjusted for malocclusion measured by PAR and the other variables in ordinal regression.

## Results

A total of 436 subjects participated in the 15-year-old survey; 364 of them (186 girls, 178 boys) were eligible for this study. Of the 73 subjects who were excluded from this study, 8 were systemically unhealthy; 65 were with orthodontic history or without oral impressions. Of the 364 eligible subjects, 331 (172 girls and 159 boys) were followed up from age 12.

Kappa value for CPI was 0.79; weighted kappa for IOTN (DHC) and IOTN (AC) were 0.92 and 0.79; ICC for DAI score, ICON score and DMFT were 0.82, 0.82 and 0.99.

Missing data only existed in some questions of family information_._ Around 30 subjects had missing data. The tackling method was as follows: if the missing data were available at age 12, the corresponding data were used in this study. As such, only 3 subjects had missing data in one or two questions, which were filled with the mode of the item data.

Table 1 presents the oral health status of subjects. No differences were found between girls and boys, except for caries. The prevalence of caries was higher in girls than in boys (*P* = 0.013). In this 15-year-old cohort, the mean DMFT (SD) was 1.70 (2.38) and the SiC index value (SD) was 4.48 (2.24). Unhealthy periodontal conditions were more prevalent than caries (92.6% vs. 52.7%). The prevalence of orthodontic treatment need was 46.7% measured by IOTN (DHC), 20.3% by IOTN (AC), 58.0% by DAI, 33.8% by ICON, and 48.4% by PAR.

**Table 1:** Profile of 15-year-old participants

	Female		Male		Total		*P*
	*N*	Percentage	*N*	Percentage	*N*	Percentage	
IOTN (DHC) treatment need							
No need	88	47.3%	106	59.6%	194	53.3%	0.052
Borderline need	48	25.8%	30	16.9%	78	21.4%	
Definite need	50	26.9%	42	23.6%	92	25.3%	
IOTN (AC) treatment need							
No need	148	79.6%	142	79.8%	290	79.7%	0.850
Borderline need	23	12.4%	27	15.2%	50	13.7%	
Definite need	15	8.1%	9	5.1%	24	6.6%	
DAI severity and treatment need							
Normal or minor malocclusion-no treatment need or slight need	78	41.9%	75	42.1%	153	42.0%	0.877
Definite malocclusion-treatment selective	55	29.6%	56	31.5%	111	30.5%	
Severe malocclusion-treatment highly desirable	36	19.4%	29	16.3%	65	17.9%	
Very severe (handicapping) malocclusion-treatment mandatory	17	9.1%	18	10.1%	35	9.6%	
ICON treatment need							
No	117	62.9%	124	69.7%	241	66.2%	0.173
Yes	69	37.1%	54	30.3%	123	33.8%	
ICON complexity							
Easy	52	28.0%	51	28.7%	103	28.3%	0.730
Mild	95	51.1%	93	52.2%	188	51.6%	
Moderate	19	10.2%	15	8.4%	34	9.3%	
Difficult	9	4.8%	12	6.7%	21	5.8%	
Very difficult	11	5.9%	7	3.9%	18	4.9%	
PAR							
Almost ideal occlusion	37	19.9%	32	18.0%	69	19.0%	0.428
Acceptable occlusion	53	28.5%	66	37.1%	119	32.7%	
Malocclusion	96	51.6%	80	44.9%	176	48.4%	
Periodontal status							
CPI score = 0	17	9.1%	10	5.6%	27	7.4%	0.201
CPI score > 0	169	90.9%	168	94.4%	337	92.6%	
CPI score < 2	35	18.8%	26	14.6%	61	16.8%	0.283
CPI score = 2	151	81.2%	152	85.4%	303	83.2%	
Caries experience							
< SiC Index value	158	84.9%	159	89.3%	317	87.1%	0.214
> =SiC Index value	28	15.1%	19	10.7%	47	12.9%	
DMFT = 0	76	40.9%	96	53.9%	172	47.3%	0.013*
DMFT> 0	110	59.1%	82	46.1%	192	52.7%	
DMFT		Mean(SD)		Mean(SD)		Mean(SD)	
	186	2.01 (2.57)	178	1.38 (2.12)	364	1.70 (2.38)	0.012*

P: comparison for DMFT used the independent samples t test; others used the Mann-Whitney U test

*IOTN* index of orthodontic treatment need, *DHC* dental health component, *AC* aesthetic component, *ICON* index of complexity, outcome and need, *PAR* peer assessment rating, *CPI* community periodontal index, *DMFT* decayed, missing and filled teeth, SiC *Index* significant caries index; SiC index value (SD) was 4.48 (2.24)

*: *P* < 0.05, **P < 0.01

Table 2 presents the results of bivariate analyses. Compared with girls, boys had lower scores in the domains of EWB, SWB and the total CPQ. Mother’s education showed some effect on the domain of OS. Subjects with unhealthy periodontal conditions had higher scores in all domains of CPQ_11–14_; however, statistical analysis showed that only subjects with CPI scores of 2 had higher scores in the domain of SWB and the total CPQ (*P* < 0.05). Subjects with a higher caries experience had higher CPQ scores in all domains of CPQ_11–14_, except for FL; nevertheless, no significant result was detected by statistical analysis. When malocclusion was classified into two groups by PAR, subjects with malocclusion had higher domains and total CPQ scores than those without malocclusion. In addition, mainly PAR detected significant results; the effects mainly existed in the domains of FL, SWB, and the total CPQ (*P* < 0.05, Table 2).

**Table 2:** Bivariate analysis between the factors and the CPQ_11–14_

		OS			FL			EWB			SWB			CPQ_11–14_ total score		
	*N*	Mean (SD)	Median (IQR)	*P*	Mean (SD)	Median (IQR)	*P*	Mean (SD)	Median (IQR)	*P*	Mean (SD)	Median (IQR)	*P*	Mean (SD)	Median (IQR)	*P*
Gender																
F	186	7.35(2.87)	7.00(4)	0.168	5.03(4.02)	4.50(5)	0.202	6.38(5.70)	5.00(8)	0.000**	3.73(4.63)	2.00(5)	0.008**	22.48(14.15)	19.00(18)	0.022*
M	178	7.70(3.05)	8.00(5)		4.49(3.81)	4.00(6)		4.48(5.35)	2.00(8)		3.14(4.72)	1.00(4)		19.81(14.03)	16.00(18)	
Total	364	7.52(2.96)	8.00(5)		4.77(3.92)	4.00(5)		5.45(5.61)	3.50(8)		3.44(4.68)	2.00(4)		21.18(14.13)	18.00(19)	
Father’s education																
Primary school graduate or below	58	7.76(3.02)	8.00(4)	0.843	5.19(4.17)	3.00(6)	0.704	6.86(6.51)	5.00(10)	0.258	4.19(5.30)	2.00(6)	0.335	24.00(15.46)	19.50(22)	0.293
Secondary school graduate or below	239	7.48(2.85)	8.00(3)		4.76(3.96)	4.00(6)		5.26(5.51)	3.00(8)		3.33(4.45)	2.00(4)		20.82(13.92)	17.00(18)	
College graduate or above	67	7.46(3.31)	8.00(5)		4.45(3.59)	4.00(4)		4.91(4.95)	3.00(7)		3.16(4.91)	1.00(5)		19.99(13.58)	16.00(17)	
Mother’s education																
Primary school graduate or below	57	8.07(3.03)	8.00(5)	0.035*	4.82(3.93)	4.00(6)	0.509	6.40(6.08)	5.00(9)	0.356	3.54(4.63)	2.00(5)	0.675	22.84(14.33)	18.00(20)	0.270
Secondary school graduate or below	264	7.33(2.90)	7.00(4)		4.70(3.91)	4.00(6)		5.27(5.57)	3.00(8)		3.48(4.85)	2.00(4)		20.78(14.34)	17.00(19)	
College graduate or above	43	7.98(3.18)	8.00(6)		5.09(4.09)	4.00(5)		5.30(5.15)	3.00(8)		3.02(3.65)	2.00(5)		21.40(12.64)	19.00(16)	
Household income																
Below HK$10,000	52	8.27(2.71)	8.00(4)	0.057	4.96(4.43)	3.00(7)	0.395	5.73(5.80)	4.00(8)	0.865	3.42(4.77)	1.00(4)	0.932	22.38(15.07)	18.00(20)	0.880
HK$10,001-HK$20,000	149	7.20(2.92)	7.00(4)		4.38(3.63)	4.00(5)		5.72(5.70)	4.00(8)		3.36(4.15)	2.00(4)		20.65(13.35)	17.00(18)	
HK$20,001-HK$30,000	61	7.34(3.02)	7.00(5)		4.70(4.04)	4.00(5)		5.48(6.11)	3.00(10)		3.48(4.96)	1.00(5)		21.00(15.60)	18.00(20)	
HK$30,001-HK$40,000	39	8.51(2.99)	9.00(5)		5.69(3.83)	6.00(7)		5.18(5.70)	2.00(8)		3.64(5.71)	1.00(5)		23.03(15.25)	16.00(18)	
Over HK$40,001	63	7.21(3.04)	7.00(4)		5.03(4.08)	4.00(6)		4.71(4.68)	3.00(9)		3.49(4.94)	2.00(5)		20.44(13.23)	19.00(18)	
Periodontal status																
CPI score = 0	27	6.63(2.75)	6.00(4)	0.095	4.26(4.48)	2.00(6)	0.247	4.85(5.97)	3.00(5)	0.533	2.11(2.74)	1.00(4)	0.285	17.85(12.28)	14.00(17)	0.209
CPI score > 0	337	7.59(2.97)	8.00(5)		4.81(3.88)	4.00(5)		5.50(5.58)	4.00(8)		3.55(4.79)	2.00(5)		21.44(14.25)	18.00(18)	
CPI score < 2	61	6.92(2.74)	6.00(4)	0.083	4.43(4.16)	3.00(5)	0.287	4.34(5.04)	3.00(7)	0.114	2.20(3.65)	1.00(3)	0.024*	17.89(11.68)	15.00(16)	0.064
CPI score = 2	303	7.64(2.99)	8.00(4)		4.84(3.88)	4.00(5)		5.67(5.69)	4.00(8)		3.69(4.83)	2.00(5)		21.84(14.50)	18.00(19)	
Caries experience																
< SiC Index value	317	7.51(3.00)	8.00(5)	0.814	4.84(3.93)	4.00(5)	0.349	5.42(5.60)	3.00(8)	0.968	3.38(4.55)	2.00(4)	0.590	21.14(13.99)	18.00(19)	0.968
> =SiC Index value	47	7.60(2.72)	8.00(4)		4.32(3.89)	3.00(5)		5.64(5.73)	4.00(9)		3.85(5.53)	2.00(5)		21.40(15.24)	16.00(17)	
IOTN (DHC) treatment need																
No need	194	7.69(3.02)	8.00(5)	0.209	4.49(4.05)	3.00(6)	0.146	5.39(5.59)	4.00(9)	0.900	3.31(4.69)	1.00(4)	0.234	20.88(14.33)	18.00(18)	0.847
Borderline need	78	7.06(2.84)	7.00(4)		5.19(3.94)	4.00(5)		5.56(5.87)	3.50(9)		3.99(4.95)	2.00(5)		21.81(14.71)	15.00(22)	
Definite need	92	7.55(2.94)	7.00(4)		4.99(3.62)	4.50(6)		5.48(5.47)	3.00(9)		3.25(4.41)	1.50(4)		21.27(13.33)	17.50(18)	
IOTN (AC) treatment need																
No need	290	7.39(2.95)	7.00(4)	0.203	4.70(3.97)	4.00(6)	0.301	5.26(5.53)	3.00(8)	0.468	3.41(4.64)	1.00(5)	0.575	20.76(14.04)	17.00(18)	0.388
Borderline need	50	8.06(2.96)	8.00(4)		4.80(4.00)	4.00(5)		6.00(5.91)	4.00(10)		3.74(5.17)	2.00(4)		22.60(15.42)	18.50(21)	
Definite need	24	8.00(3.09)	8.50(6)		5.54(3.20)	5.00(5)		6.54(5.95)	5.00(12)		3.17(4.26)	2.00(4)		23.25(12.65)	19.00(20)	
DAI																
Normal or minor malocclusion-no treatment need or slight need	153	7.31(2.98)	7.00(5)	0.049*	4.35(3.80)	4.00(6)	0.176	5.07(5.17)	3.00(9)	0.214	3.17(4.31)	1.00(4)	0.088	19.91(13.29)	18.00(18)	0.060
Definite malocclusion-treatment selective	111	7.89(3.20)	8.00(4)		5.06(4.15)	4.00(6)		5.79(6.01)	4.00(9)		4.11(5.36)	2.00(6)		22.86(15.74)	18.00(22)	
Severe malocclusion-treatment highly desirable	65	6.94(2.67)	6.00(4)		4.78(3.93)	3.00(6)		5.02(5.95)	3.00(7)		2.66(4.13)	1.00(4)		19.40(13.56)	14.00(17)	
Very severe (handicapping) malocclusion-treatment mandatory	35	8.31(2.37)	8.00(4)		5.63(3.61)	5.00(5)		6.80(5.43)	7.00(9)		3.94(4.72)	2.00(4)		24.69(12.64)	23.00(17)	
ICON treatment need																
No	241	7.40(3.05)	7.00(5)	0.319	4.55(3.97)	4.00(6)	0.061	5.29(5.62)	3.00(9)	0.291	3.51(4.75)	2.00(5)	0.519	20.74(14.42)	17.00(19)	0.207
Yes	123	7.76(2.77)	8.00(4)		5.20(3.81)	5.00(6)		5.76(5.58)	4.00(9)		3.31(4.56)	2.00(4)		22.02(13.57)	18.00(19)	
ICON complexity																
Easy	103	7.41(3.11)	8.00(5)	0.329	3.94(3.66)	3.00(5)	0.079	4.70(5.26)	2.00(8)	0.300	2.78(4.12)	1.00(4)	0.306	18.83(13.17)	16.00(18)	0.213
Mild	188	7.36(2.89)	7.00(4)		5.06(4.05)	4.00(6)		5.49(5.58)	4.00(8)		3.89(5.11)	2.00(5)		21.80(14.60)	18.00(18)	
Moderate	34	7.97(3.02)	8.00(4)		5.06(4.61)	4.00(9)		6.00(6.36)	3.00(11)		2.88(4.03)	1.00(4)		21.91(15.51)	15.50(26)	
Difficult	21	8.52(2.70)	9.00(4)		5.00(2.57)	5.00(4)		6.71(5.64)	5.00(9)		3.19(3.99)	2.00(5)		23.43(11.95)	20.00(16)	
Very difficult	18	7.78(3.04)	8.50(5)		5.61(3.53)	5.00(5)		6.78(6.25)	6.00(12)		3.89(4.68)	2.00(3)		24.06(13.81)	21.50(26)	
PAR																
Almost ideal or Acceptable occlusion	188	7.38(3.15)	7.00(5)	0.397	4.30(3.87)	3.00(5)	0.010*	5.08(5.60)	3.00(9)	0.078	3.16(4.55)	1.00(4)	0.049*	19.93(14.19)	17.00(18)	0.031*
Malocclusion	176	7.66(2.75)	8.00(4)		5.27(3.93)	4.50(6)		5.84(5.60)	4.00(9)		3.73(4.81)	2.00(5)		22.51(13.99)	18.00(18)	

Nonparametric tests: comparison between two samples used the Mann-Whitney U test; others used the Kruskal-Wallis H test; *: *P* < 0.05, **P < 0.01

*IOTN* index of orthodontic treatment need, *DHC* dental health component, *AC* aesthetic component, *ICON* index of complexity, outcome and need, *PAR* peer assessment rating, *CPI* community periodontal index, *DMFT* decayed, missing and filled teeth

SiC *Index* significant caries index, *OS* oral symptoms domain, *FL* functional limitations domain, *EWB* emotional well-being, *SWB* social well-being, *CPQ* child perceptions questionnaire, *SD* standard deviation, *IQR* interquartile range

Table 3 presents the results of ordinal regression. The results of gender, family ecosocial factors, periodontal status and caries were almost the same with the bivariate analyses. Compared with girls, boys tended to have a better experience in the domains of EWB, SWB, and the total CPQ (adjusted OR = 0.46, 0.59 and 0.61, respectively). Take the total CPQ for example. Boys had 0.61 times the likelihood of having a higher rank when compared with girls (*P* = 0.011). Little effect of family ecosocial factors and caries was found by regression analysis. As for periodontal status, CPI scores of 2 had a negative effect on the domain of SWB and the total CPQ (adjusted OR = 1.76 and 1.71, respectively). A more severe level of malocclusion was associated with a higher likelihood of having a higher rank in all domains of CPQ_11–14_. However, only the domain of FL and the total CPQ were affected by the most severe malocclusion; mainly PAR and DAI detected the significant effect. For example, when compared with “the no/minor” malocclusion group measured by DAI, only “the very severe” malocclusion was associated with a higher likelihood of having a higher rank in the total CPQ after adjusting the effects of other factors (adjusted OR = 2.10, *P* = 0.032).

**Table 3:** Ordinal regression of associations between the factors and the CPQ_11–14_

	OS		FL		EWB		SWB		CPQ_11–14_	
	Adjusted OR (95%CI)	*P*	Adjusted OR (95%CI)	*P*	Adjusted OR (95%CI)	*P*	Adjusted OR (95%CI)	*P*	Adjusted OR (95%CI)	*P*
Sociodemographic status										
Gender										
F^a^										
M	1.43 (0.97, 2.10)	0.069	0.76 (0.52, 1.12)	0.172	0.46 (0.31, 0.67)	0.000**	0.59 (0.40, 0.87)	0.008**	0.61 (0.41, 0.89)	0.011*
Father’s education										
Primary school graduate or below^a^										
Secondary school graduate or below	1.01 (0.56, 1.82)	0.963	0.85 (0.47, 1.52)	0.582	0.89 (0.50, 1.60)	0.694	0.81 (0.45, 1.45)	0.477	0.70 (0.39, 1.26)	0.234
College graduate or above	0.75 (0.35, 1.64)	0.476	0.57 (0.26, 1.25)	0.161	0.97 (0.45, 2.11)	0.940	0.58 (0.26, 1.27)	0.170	0.52 (0.24, 1.14)	0.101
Mother’s education										
Primary school graduate or below^a^										
Secondary school graduate or below	0.64 (0.36, 1.15)	0.137	0.86 (0.48, 1.54)	0.610	0.88 (0.49, 1.58)	0.666	0.88 (0.49, 1.58)	0.666	0.90 (0.50, 1.61)	0.723
College graduate or above	1.46 (0.60, 3.55)	0.407	1.10 (0.45, 2.69)	0.836	0.91 (0.37, 2.22)	0.833	1.35 (0.55, 3.31)	0.510	1.41 (0.58, 3.42)	0.451
Household income										
Below HK$10,000^a^										
HK$10,001-HK$20,000	0.65 (0.36, 1.17)	0.151	1.03 (0.57, 1.84)	0.934	1.14 (0.64, 2.05)	0.659	1.28 (0.71, 2.31)	0.403	0.95 (0.53, 1.69)	0.855
HK$20,001-HK$30,000	0.66 (0.33, 1.32)	0.241	1.06 (0.53, 2.12)	0.878	1.04 (0.52, 2.09)	0.906	1.29 (0.64, 2.60)	0.474	1.06 (0.53, 2.11)	0.879
HK$30,001-HK$40,000	1.34 (0.62, 2.88)	0.463	2.37 (1.09, 5.16)	0.030*	0.93 (0.43, 2.03)	0.861	1.10 (0.50, 2.39)	0.818	1.30 (0.60, 2.81)	0.501
Over HK$40,001	0.61 (0.29, 1.31)	0.204	1.75 (0.82, 3.76)	0.150	1.04 (0.49, 2.23)	0.921	1.37 (0.64, 2.94)	0.421	1.12 (0.53, 2.39)	0.763
Periodontal and caries status										
Periodontal status										
CPI score < 2^a^										
CPI score = 2	1.34 (0.80, 2.24)	0.261	1.26 (0.76, 2.11)	0.372	1.48 (0.89, 2.48)	0.132	1.76 (1.05, 2.96)	0.033*	1.71 (1.03, 2.85)	0.038*
Caries experience										
< SiC Index value^a^										
> =SiC Index value	1.05 (0.60, 1.85)	0.864	0.63 (0.35, 1.12)	0.112	0.79 (0.45, 1.40)	0.425	1.17 (0.66, 2.06)	0.585	0.95 (0.54, 1.66)	0.850
Malocclusion										
IOTN (DHC) treatment need										
No need^a^										
Borderline need	0.60 (0.37, 0.99)	0.047*	1.28 (0.78, 2.09)	0.327	1.04 (0.63, 1.70)	0.888	1.45 (0.88, 2.37)	0.142	0.97 (0.60, 1.58)	0.905
Definite need	1.03 (0.66, 1.63)	0.887	1.31 (0.83, 2.06)	0.250	1.07 (0.68, 1.68)	0.778	1.15 (0.73, 1.81)	0.556	1.08 (0.69, 1.69)	0.744
IOTN (AC) treatment need										
No need^a^										
Borderline need	1.60 (0.92, 2.78)	0.096	1.00 (0.57, 1.73)	0.987	1.45 (0.84, 2.53)	0.186	1.31 (0.76, 2.29)	0.333	1.32 (0.76, 2.29)	0.321
Definite need	1.66 (0.77, 3.56)	0.197	1.88 (0.87, 4.06)	0.107	1.44 (0.67, 3.11)	0.348	0.98 (0.45, 2.11)	0.955	1.34 (0.62, 2.88)	0.452
DAI severity and treatment need										
Normal or minor malocclusion-no treatment need or slight need^a^										
Definite malocclusion-treatment selective	1.28 (0.82, 2.01)	0.281	1.40 (0.89, 2.21)	0.141	1.29 (0.82, 2.03)	0.265	1.54 (0.98, 2.43)	0.060	1.34 (0.86, 2.10)	0.199
Severe malocclusion-treatment highly desirable	0.82 (0.48, 1.40)	0.462	1.05 (0.61, 1.80)	0.859	0.87 (0.51, 1.50)	0.623	0.68 (0.39, 1.17)	0.162	0.76 (0.45, 1.30)	0.316
Very severe (handicapping) malocclusion-treatment mandatory	1.62 (0.83, 3.19)	0.159	1.93 (0.98, 3.79)	0.057	1.85 (0.94, 3.63)	0.076	1.47 (0.75, 2.89)	0.263	2.10 (1.06, 4.13)	0.032*
ICON treatment need										
No^a^										
Yes	1.23 (0.82, 1.83)	0.318	1.40 (0.94, 2.09)	0.101	1.22 (0.82, 1.82)	0.329	1.10 (0.74, 1.65)	0.639	1.21 (0.81, 1.81)	0.343
ICON complexity										
Easy^a^										
Mild	0.77 (0.50, 1.20)	0.249	1.63 (1.04, 2.54)	0.033*	1.44 (0.92, 2.25)	0.106	1.71 (1.09, 2.67)	0.020*	1.52 (0.97, 2.36)	0.065
Moderate	1.32 (0.65, 2.69)	0.443	1.38 (0.68, 2.82)	0.375	1.42 (0.70, 2.91)	0.333	1.38 (0.67, 2.82)	0.382	1.54 (0.76, 3.13)	0.232
Difficult	1.59 (0.67, 3.78)	0.294	2.08 (0.87, 4.96)	0.101	2.61 (1.09, 6.27)	0.031*	1.56 (0.65, 3.73)	0.315	2.16 (0.91, 5.13)	0.082
Very difficult	1.35 (0.54, 3.39)	0.523	2.71 (1.07, 6.86)	0.035*	2.00 (0.79, 5.05)	0.141	1.94 (0.77, 4.90)	0.160	1.90 (0.76, 4.77)	0.172
PAR score range										
Almost ideal or Acceptable occlusion ^a^										
Malocclusion	1.15 (0.79, 1.69)	0.457	1.55 (1.06, 2.28)	0.023*	1.38 (0.94, 2.01)	0.099	1.41 (0.96, 2.06)	0.078	1.35 (0.92, 1.97)	0.121

Statistical method: Ordinal regression (link function: logit; model: main effects), each orthodontic index adopted one separate ordinal regression; dependent variable: CPQ scores classified into four groups with cut-off points as quartile (1: scores < = first quartile; 2: first quartile < scores < = second quartile; 3: second quartile < scores < = third quartile; 4: scores > third quartile); ^a^: reference group; *: *P* < 0.05. **: *P* < 0.01

N: sample size; adjusted OR: malocclusions adjusted for gender, father’s education level (primary school graduate or below; secondary school, post-secondary or above), mother’s education level (levels set as father’s education), household income (Below HK$10000, HK$10001-HK$20000, HK$20001-HK$30000, HK$30001-HK$40000, HK$40001 or above), caries experience (DMFT < SiC Index value, DMFT > = SiC Index value), and periodontal status (CPI score < 2, CPI score = 2); gender, socioeconomic status, periodontal and caries status adjusted for the previous variables and malocclusion measured by PAR (almost ideal occlusion, acceptable occlusion, malocclusion)

*IOTN* index of orthodontic treatment need, *DHC* dental health component, *AC* aesthetic component, *ICON* index of complexity, outcome and need, *PAR* peer assessment rating; *CPI* community periodontal index, *DMFT* decayed, missing and filled teeth, SiC *Index* significant caries index, *OS* oral symptoms domain, *FL* functional limitations domain, *EWB* emotional well-being domain, *SWB* social well-being domain, *CPQ* child perceptions questionnaire, *OR* odds ratio, *CI* confidence interval

## Discussion

This cross-sectional study analysed the influence factors of OHRQoL based on a representative sample of 15-year-old children. The prevalence of caries was higher in girls than in boys. Gender, periodontal status and malocclusion, but not family ecosocial factors and caries, could have an effect on OHRQoL. Boys might be more likely to have a better experience in the domains of EWB, SWB and the total CPQ. Unhealthy periodontal conditions could have a worse effect on SWB and the total CPQ. Only the most severe level of malocclusion could have a significant effect on FL and the total CPQ.

There were some differences when comparing the results at age 15 with those at age 12 [34]. First, the oral health status of girls was not different from boys at age 12; while girls had more caries than boys at age 15. Additionally, boys might have a worse experience in the domain of OS but a better experience in EWB at age 12; while boys might have better experiences in EWB, SWB and total CPQ at age 15. This indicated that boys had more positive experiences of OHRQoL than girls did. Second, at age 12, household income did not show an effect on OHRQoL after correcting other factors. Mother’s education could have a positive effect, while father’s education could have a negative effect on children’s OHRQoL. However, at age 15, these family factors showed little effect on OHRQoL. These results suggested subjects at different age may have different experiences of OHRQoL, which supported the hypothesis that quality of life is a “dynamic construct” that is likely to change with age [4]. Therefore, age should be considered as a predictor of OHRQoL, and the cut-off points of age periods should be investigated. It was suggested that subjects’ OHRQoL was less likely to be impacted at age 15–18 years than at age 12–15 years; the influence of age was further reduced and became stable above 18 years old [35]. However, these results were only from cross-sectional studies. To date no longitudinal study has been conducted to follow subjects from 12 to 18 years old.

Unhealthy periodontal conditions were more prevalent than caries in both this study and the study at age 12. At age 12 the effect of unhealthy periodontal conditions was detected on the domain of EWB and the total CPQ, while at age 15 the effect was on SWB and the total CPQ. A cross-sectional study from Brazil with a sample of 286 schoolchildren of 12 years old also reported that the presence of bleeding had an impact on the domains of EWB and SWB of CPQ_11–14_ [9]. However, when 170 children were followed from age 12 to 15, the presence of bleeding showed no impact on OHRQoL [36].

Some studies reported caries had an impact on OHRQoL [36, 37], while other studies denied it [9, 38]. In this study, high caries experience showed no effect on OHRQoL at age 15. These results seemed to support the hypothesis that health problems may affect quality of life, but such a consequence is not inevitable [3]. The possible explanation is that Hong Kong is an economically developed area with mature preventive and treatment conditions for caries; thus the symptoms of caries are not likely to hazard children’s OHRQoL.

Many studies reported the negative impact of untreated malocclusion on CPQ_11–14_ scores [9, 39–41]; nevertheless, there are also studies reporting malocclusion had no impact on CPQ_11–14_ scores [42, 43]. In this study, malocclusion showed an impact on all domains of CPQ_11–14_ except for OS at age 12; while it only showed an impact on the domain of FL and the total CPQ_11–14_ at age 15; both studies showed that only severe malocclusions had effects on OHRQoL. A study from Brazil reported that orthodontic treatment need had a strong negative effect on the domain of FL in 12-year-old children [9]. Another study from Saudi Arabia also showed only children with very severe (handicapping) malocclusion had significantly higher domain and total CPQ_11–14_ scores than all the other groups of 11 to 14 years old children [40].

The results generated by orthodontic indices were different between the two studies, too. At age 12, all indices detected the effects of malocclusion on some domains of CPQ_11–14_ [34]; while at age 15, only PAR and DAI detected the effect of malocclusion on the domain of FL and the total CPQ. The ability of PAR in detecting the effect of malocclusion on FL was confirmed by both studies at age 12 and age 15. Therefore, PAR may be a good index to judge the effect of malocclusion on subjects’ oral functional limitations, such as breathing through mouth, taking longer to eat a meal, having difficulties to open mouth wide or chew firm foods. PAR is an index that evaluates all anomalies that constitute a malocclusion, while other indices either put great weight on dental esthetics, i.e., the frontal aspects of malocclusion, or is based on the most severe malocclusion trait, like IOTN (DHC) [25–28]. Thus the functional limitations caused by malocclusion can be more easily detected by PAR in this study, or by both PAR and IOTN (DHC) in the study of age 12. Other orthodontic indices may be more suitable to assess the effect of malocclusion on subjects’ social lives, as showed in the study of age 12.

CPQ_11–14_ was designed for children of 11 to 14 years old. In this study, CPQ_11–14_ was used to measure OHRQoL of 15-year-old children, so that the data of this study could be compared with the data of 12-year-old children. There are other studies using CPQ_11–14_ to measure OHRQoL of children older than 14 years old [36, 44, 45]. All variables in this study were included into ordinal regression and the main effect models were used to calculate adjusted ORs. This cannot waive the potential of interaction between variables, though in this study the significant results of multivariate analyses were almost the same with the bivariate analyses.

When looking into the disadvantages of this study, four aspects should be considered. First, although this article was based on a representative sample, given that this is only a cross-sectional analysis, the results should be treated with caution. Further longitudinal analysis of this research should provide more definitive evidences. Second, this study was part of a longitudinal study planning to follow subjects from age 12 to 18. Sample size in the original design was calculated [46, 47] based on a previous study [10]. With a lost rate of 30% at each follow-up and the design effect for cluster sampling considered, the sample sizes at ages 12, 15, and 18 should be 237, 166, and 116, respectively [34]. This calculation was for the longitudinal study, not for this cross-sectional study. In addition, the sample size estimation was based on the method of comparing two means. Although the design effect of cluster randomized trials with unequal cluster has been examined, the effect of the statistical methods should still be considered. Third, all variables were included into multi-factor analyses of ordinal regression. Although the single-factor analyses of ordinal regression were also performed during data analysis, which showed almost the same results with the multi-factor analyses, the interpretation of the results should still be treated with caution. Fourth, the sample of this study was only selected in Hong Kong; studies from other regions or countries may disagree with this study because of different geographical, cultural, and economical situations.

## Conclusion

The influence factors of OHRQoL were studied based on a representative sample of 15-year-old children. Boys were less likely to have caries and more likely to have better OHRQoL than girls were. Family ecosocial factors and caries showed little effect on OHRQoL. Unhealthy periodontal conditions could lead to worse social well-beings and OHRQoL. The most severe level of malocclusion could cause functional limitations, and hence a worse OHRQoL.

## Abbreviations

AC: Aesthetic component
CPI: Community Periodontal Index
CPQ: Child perceptions questionnaire
DAI: Dental aesthetic index
DHC: Dental health component
DMFT: Decayed, Missing and Filled Teeth
EWB: Emotional well-being
FL: Functional limitations domain
ICON: Index of complexity, outcome and need
IOTN: Index of orthodontic treatment need
OHRQoL: Oral health-related quality of life
OR: Odds ratio
OS: Oral symptoms domain
PAR: Peer assessment rating
SD: Standard deviation
SE: Standard error
SiC index: Significant caries index
SWB: Social well-being
WHO: World Health Organization
